# Hypocholesterolemic effects of Balangu (*Lallemantia royleana*) seeds in the rabbits fed on a cholesterol-containing diet

**Published:** 2015

**Authors:** Alireza Ghannadi, Ahmad Movahedian, Zahra Jannesary

**Affiliations:** 1 *Department of Pharmacognosy, School of Pharmacy and Pharmaceutical Sciences, Isfahan University of Medical Sciences, Isfahan, Iran*; 2 *Department of Biochemistry, School of Pharmacy and Pharmaceutical Sciences, Isfahan Pharmaceutical Sciences Research Centre, Isfahan University of Medical Sciences, Isfahan, Iran *

**Keywords:** *Lallemantia royleana Lamiaceae*, *Iranian Traditional Medicine*, *Cholesterol*, *Hypercholesterolemia*

## Abstract

**Objectives:**

*Lallemantia royleana *(Benth. in Wall.) Benth. (Lamiaceae) is a medicinal plant used in Iranian traditional and folklore medicine in the treatment of various nervous, hepatic, and renal diseases. In the present study, whole seeds of the herb were prepared and evaluated for hypolipidemic activities using an animal model.

**Materials and Methods:**

Animals were fed normal diets or diets supplemented with cholesterol (0.5%) for 12 weeks to evoke hypercholesterolemia. Moreover, hypercholesterolemic animals were treated with different doses of whole seeds of Balangu (0, 5, 10, and 20%) for 12 weeks.

**Results:**

Results showed that the serum total cholesterol and triglyceride decreased in all groups treated with Balangu seeds p<0.05. Changes in the distribution of cholesterol in low density lipoprotein cholesterol (LDL-C) and high density lipoprotein cholesterol (HDL-C) were found. LDL-C and HDL-C decreased significantly in all of the groups treated with whole seeds of the herb with respect to hypercholesterolemic group p<0.05.

**Conclusion:**

Our results showed that *L. royleana* seeds decreased the serum cholesterol and triglyceride levels in hypercholesterolemic animals but led to the increase of atherogenic index in all treated groups.

## Introduction

Finding natural, safe, and effective treatments for dyslipidemia and hyperlipidemia is one of the major quests of medical scientists. Medicinal, functional, and nutraceutical herbs have been used as food and for medicinal purposes for centuries. Research interests have focused on various herbs that possess hypolipidemic and hypocholesterolemic properties that may be useful for reducing the risk of cardiovascular diseases (Kanakavalli et al., 2014 ▶; Movahedian et al., 2007 ▶). Dragon head or* Lallemantia royleana *(Benth. in Wall.) Benth. is one of the Iranian medicinal plants that belongs to the Lamiaceae family. It is one of the five species of Iranian *Lallemantia* which grows wild in several areas of the country (North, East North, East South, and Alborz Mountains) (Mozaffarian, 1996 ▶; Samadi et al., 2007 ▶). *Lallemantia* species can be used for a variety of purposes including lightening, varnish, painting, food, and medicine. The genus is also distributed in Afghanistan, Pakistan, India, China, Syria, Iraq, Turkmenistan, Tajikistan, Kyrgyzstan, Kazakhstan, Uzbekistan, Russia, and some other European countries (Samadi et al., 2007 ▶; Dinc et al., 2009 ▶). These aromatic herbs commonly known in Iran as “Balangu“ or “Balangoo”, “Balangu Shahri”, and “Balangu Shirazi” (Samadi et al., 2007 ▶; Amin, 2005 ▶; Ghannadi and Zolfaghari, 2003 ▶). Seeds of *L. royleana* are dark-brown to black in color and smooth. When moistened with water, the seeds become coated with translucent and voluminous mucilage and hydrocolloids. The taste of the moistened seed is bland, soothing, and spicy (Malavya and Dutt, 1941 ▶; Mohammad Amini and Razavi, 2012 ▶). Its mucilage can be used for application in food and drink industries such as ice cream and natural gel making (Bahramparvar et al., 2008 ▶; Abdulrasool et al., 2011 ▶). 

The seeds have been used in Iranian traditional and folklore medicine as diuretic, tonic, aphrodisiac, and anti-tussive remedy and in the treatment of various nervous, hepatic, and renal disorders (Amin, 2005 ▶; Ghannadi and Zolfaghari, 2003 ▶). Seeds powder have been used in some southern parts of Iran as a tonic medication and a remedy for psychotic diseases (Safa et al., 2013 ▶). The seeds have been added to a variety of Persian foods, drinks, and sherbets for flavoring, cooling, and soothing. 

A few reports on the analysis of the seeds of *L. royleana *and other *Lallemantia *species have been published. These species contain some similar biologically active compounds such as polysaccharides, soluble fibers and mucilages, fixed oils, proteins, and essential oils (Samadi et al., 2007 ▶; Ghannadi and Zolfaghari, 2003 ▶; Baser et al., 2000 ▶; Marin et al., 191 ▶; Fekri et al., 2008 ▶; Razavi et al., 2008 ▶; Fekri et al., 2008 ▶; Amanzadeh et al., 2011 ▶). Recently, antibacterial activities of *L. royleana *seeds were reported (Mahmood et al., 2013 ▶). In some regions of Iran, it is claimed to be effective against hyperlipidemia.

The present study was carried out in an attempt to investigate the potential hypolipidemic effects of *L. royleana *seeds in an animal model. 

## Materials and Methods

**Plant material**

The seeds of *L. royleana* were purchased from local market of Isfahan, Iran. The seeds were cultivated in pods in the Botanical Garden of Isfahan University of Medical Sciences. A voucher herbarium specimen was prepared for necessary systematic identification. The name of *L. royleana* was confirmed by the herbarium department of Islamic Azad University, Science and Research branch, Tehran, Iran. This voucher specimen was deposited in the herbarium of School of Pharmacy and Pharmaceutical Sciences, Isfahan University of Medical Sciences, Isfahan, Iran for future evidence.

**Diet and treatment**

Twenty five male New Zealand white rabbits weighing about 1.5 kg on arrival were obtained from the central animal house of the Tehran Pasteur Institute, Tehran, Iran. The animals were housed in temperature (21-23 ^°^C) and light controlled room with a 12-h light-dark cycle and ambient humidity (50-60%). All of the rabbits were initially fed a normal diet (Pars Dam, Tehran, Iran) for 2 weeks and then randomly divided into a normal control group (n=5, fed a normal diet), a hypercholesterolemic control group (n=5, fed 0.5% high cholesterol diet) and three treatment groups (n=5, fed 0.5% high cholesterol diet along with 5, 10, and 20% freshly powdered balango seeds). Animals were fed for 12 weeks and each diet was set 100 g/rabbit per day with water available ad libitum (Movahedian et al., 2006 ▶). 

**Experimental procedure**

 The rabbits were weighed weekly. Blood samples from a marginal ear vein were taken at 0, 6, and 12 weeks after 12 h of fasting to monitor any changes in the total cholesterol, triglyceride (TG), and lipoprotein cholesterol. Serum was separated by centrifugation (2000 g, 20 min, 4 ^°^C) and used for biochemical analysis. Serum cholesterol and triglyceride levels were determined using commercially available kits (Pars Azmoon, Tehran, Iran). The lipoprotein cholesterol concentrations (HDL-C and LDL-C) were assessed colorimetrically with an enzyme assay kits (Pars Azmoon, Tehran, Iran). The atherogenic index (total cholesterol – HDL cholesterol / HDL cholesterol) was also calculated.

**Statistical analysis**

All data are presented as mean ± SD. Statistical analysis of the data was done using one-way analysis of variance (ANOVA), followed by Tukey’s multiple comparisons test. Statistical significance was accepted at p<0.05.

## Results

**Figure 1:**
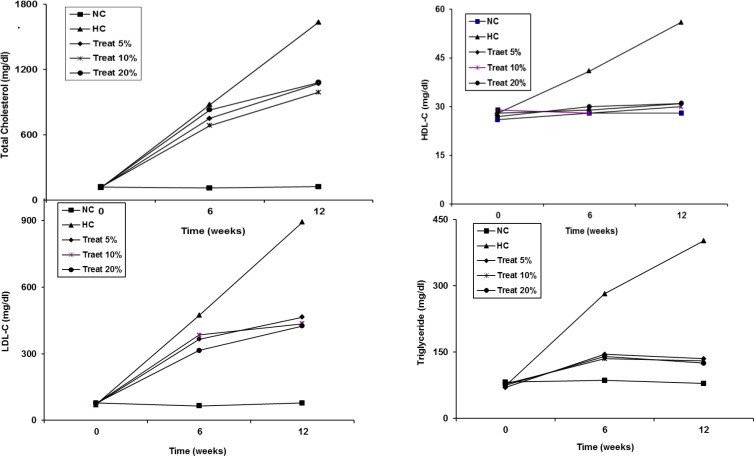
Lipid profiles at 0, 6, and 12 weeks of treatment with 5, 10, and 20% *L. royleana* seeds compared with normal control (NC) and hypercholesterolemic control (HC)**. **Data are significantly different at p<0.05 compared with HC group.

Compared to the normal control group in a period of 12 weeks, the serum levels of total cholesterol and low/high density lipoprotein cholesterols (LDL-C and HDL-C) were increased in hypercholesterolemic control group (Table 1). The effects of *L. royleana* seeds on serum lipids level in rabbits, which were fed enriched cholesterol diet, are presented in Figure 1.

The results indicated that upon *L. royleana* seeds consumption for 12 weeks, serum triglyceride, total cholesterol, LDL-C, and HDL-C decreased significantly at doses of 5, 10, and 20% of freshly powdered balango seeds compared with the hypercholesterolemic control group (Figure 1) p<0.05. However, *L. royleana* seeds showed no decrease in atheroginic index in all treatment groups with respect to the hypercholesterolemic control group (Table 2).

**Table 1:** Serum lipid profiles in cholesterol fed rabbits compare with normal control

**Group**	**Time (weeks)**	**Total cholesterol (mg/dl)**	**Triglyceride (mg/dl)**	**LDL-C (mg/dl)**	**HDL-C (mg/dl)**
**NC**	0	120 ± 19	82 ± 12	78 ± 19	26 ± 6
	12	122 ± 17	79 ± 13	78 ± 21	28 ± 7
**HC**	0	113 ± 18	74 ± 12	70 ± 18	28 ± 6
	12	1634 ± 81*	402 ± 28*	893 ± 64*	56 ± 10*

Values are means ± SD (n=5).

* Data in columns were significantly different at p<0.05. NC: normal control; HC: hypercholesterolemic control.

**Table 2:** Effect of *L. royleana* seeds on atherogenic index in cholesterol fed rabbits at 12 weeks of treatment compared with normal control (NC) and hypercholesterolemic control (HC).

**Group**	**Atherogenic index**
**NC **	3.4 ± 1.0
**HC**	28.2 ± 3.1*
***L. royleana*** ** seeds treatment groups**	
** 5% **	33.5 ±3.2
** 10% **	32.0 ±2.6
** 20% **	33.8 ± 2.9

Data are mean ± SD (n = 5).

NC: normal control; HC: hypercholesterolemic control.

* Data are significantly different at p<0.05 compared with NC group.

. 

## Discussion

With regard to the effect of dietary supplements, our results showed that addition of *L. royleana* seeds to the cholesterol-enriched diet decreased the hyperlipidemia induced by a high-cholesterol diet in rabbits. The results indicated that consumption of *L. royleana* seeds for 12 weeks significantly decreased the serum total cholesterol, LDL-C, and HDL-C at all doses compared with the hypercholesterolemic group (Figure 1). Because apo B containing lipoprotein fractions are thought to be responsible for cholesterol deposition in atherosclerotic plaques (Schaefer and Asztalos, 2006 ▶), a reduction in LDL-C would be advantageous clinically in hypercholesterolemia. It was shown that balangu seeds have an improving effect on the hypercholesterolemia induced by a high fat diet. Soluble dietary fibers such as mucilage that are capable of forming gels are main constituents of some of the spices and condiments. These substances have remarkable beneficial activities including the ability to decrease blood lipids. The hypocholestrolemic mechanisms of mucilaginous compounds of the plants are not evidently defined (Boban et al., 2009 ▶; Arjmandi et al., 1992 ▶). It was demonstrated that soluble fibers reduce serum lipid levels by reducing the rate of production of very low density lipoprotein (VLDL) by liver cells and that the hypolipidemic effect varied with the chemical nature of the soluble fiber (Boban et al., 2006 ▶). The cholesterol lowering effect of flax seed and fenugreek has been attributed to the mucilage which is present in these materials (Lucas et al., 2004 ▶; Kumar et al., 2005 ▶). The hypolipidemic effects of dietary fibers have been attributed to their ability to inhibit intestinal absorption of bile acids and neutral steroids, resulting in greater fecal bile acid and total steroid excretions (Kumar et al., 2005 ▶; Gelisse et al., 1994 ▶). *L. royleana* seed oil has high oleic, linoleic, and linolenic acid content. Their mean amounts in the oil are 17%, 17% and more than 50% respectively (Samadi et al., 2007 ▶). These types of fatty acids are known to have anti-inflammation, antiplatelet aggregation, antihypertension, and antihyperlipidemia properties (Riediger et al., 2009 ▶).* Lallemantia* seeds may act similar to flax seeds and other plant oils as a source of linolenic fatty acid (Zlatanov et al., 2012 ▶).

According to the significant and simultaneous decrease in LDL-C and HDL-C, in *L. royleana* seeds treatment groups, the level of atheroginic index does not indicate any significant decrease in these groups compared with hypercholesterolemic control group (Table 2).

Therefore, in spite of the decrease in the serum levels of total cholesterol and LDL-C in treatment groups with the considered seed, it seems difficult to judge the reducing effects of this plant, due to the lack of any reduction of atherogenic index in such groups. 

Despite observed reducing effects on serum total cholesterol and LDL-C, using this plant in Iranian folk medicine would be of question for the treatment of hyperlipidemia and further researches are required. 

## Conflict of interest

The authors declare that there are no conflicts of interest.
